# Primordial magnetotaxis in putative giant paleoproterozoic magnetofossils

**DOI:** 10.1073/pnas.2319148121

**Published:** 2024-05-28

**Authors:** Ualisson Donardelli Bellon, Wyn Williams, Ricardo Ivan Ferreira Trindade, Lara Maldanis, Douglas Galante

**Affiliations:** ^a^Department of Geophysics, Institute of Astronomy, Geophysics and Atmospheric Sciences, University of São Paulo, São Paulo 05360020, Brazil; ^b^Department of Geophysics, School of Geosciences, University of Edinburgh, Edinburgh EH9 3FE, Scotland; ^c^Earth Science Department, Vrije Universiteit Amsterdam, Amsterdam 1081 HV, the Netherlands; ^d^Department of Sedimentary and Environmental Geology, Institute of Geosciences, University of São Paulo, São Paulo 05508080, Brazil

**Keywords:** giant magnetofossils, magnetotaxis, primordial, micromagnetic modeling

## Abstract

Magnetotactic bacteria produce chains of nanoscopic iron minerals used for navigation, which can be preserved over geological timescales in the form of magnetofossils. Micrometer-sized magnetite crystals with unusual shapes suggesting a biologically controlled mineralization have been found in the geological record and termed giant magnetofossils. The biological origin and function of giant magnetofossils remains unclear, due to the lack of modern analogues to giant magnetofossils. Using distinctive Ptychographic nanotomography data of Precambrian (1.88 Ga) rocks, we recovered the morphology of micrometric cuboid grains of iron oxides embedded in an organic filamentous fossil to construct synthetic magnetosomes. Their morphology is different from that of previously found giant magnetofossils, but their occurrence in filamentous microfossils and micromagnetic simulations support the hypothesis that they could have functioned as a navigation aid, akin to modern magnetosomes.

## Significance

Microscopic organisms that capture iron from the environment and produce nanoscopic magnetic grains along their bodies are among the primordial forms of life. When organized into chain-like structures, these grains enable navigation by aligning with the Earth’s field. These ancient fossils offer insights into early life and Earth’s primordial environments. Our study examined whether unusually large iron oxide grains found in 1.9 billion-year-old fossils could replicate this orientation mechanism. The results revealed that even under weak magnetic fields, these “giant” chain structures behave as efficient magnetic compasses. Furthermore, their diagnostic signatures are sufficiently different from more common magnetic bacteria suggesting that the presence of these larger chain structures in the geological record may have been underestimated.

Biomineralization, the process of organisms crystallizing minerals inside or outside their cells (1), has been vital for Earth’s cycles since the Archean (2). One of the earliest forms of biomineralization is the synthesis of magnetosomes, arranged as chains of equant magnetite (Fe_3_O_4_) or greigite (Fe_3_S_4_) grains, within magnetotactic bacteria (MT) (3). These iron oxide or sulfide grains enabled the magnetotactic bacteria to perform magnetotaxis, helping them navigate to ideal conditions for metabolic functions (4–6). These biomineralized particles may occur in distinct morphologies, including hexagonal prisms, cuboidal, and bullet/arrow shapes (7), setting them apart from nonbiological iron oxides (8, 9). Magnetosomes are found in several different settings in modern sedimentary environments (10). They are usually formed by chains of nanometric-scaled particles, but “giant” micrometric magnetofossils with spearhead/spindle-like and hexaoctahedral prisms have been reported in Paleocene/Eocene sediments (7, 11).

Molecular clock dating suggests that magnetotactic biomineralization originated before or near the divergence between *Nitrospirae* and *Proteobacteria* phyla during the Archean (12). Nevertheless, firm fossil evidence for the presence of magnetotactic bacteria in Precambrian strata is still contentious. The earliest fossil evidence for grains produced by magnetotactic bacteria dates from 1.9 Ga (5, 13), where chains of nanoscopic magnetite particles have been recovered from magnetic extracts of rocks from the Gunflint formation. This Late Proterozoic formation extends from NE-Minnesota, USA, to the Thunder Bay area of Ontario, Canada, characterized by Fe-rich clastic/volcanoclastic sedimentary formations. It bears a diverse range of well-preserved filamentous and coccoidal fossils (14, 15), some showing fossil filaments of the *Gunflitia* genus, possibly cyanobacteria or chemotrophic bacteria that oxidized iron (14, 16–18). Fe-silicates and Fe-carbonates in microfossils suggest in vivo intracellular Fe-biomineralization with subsequent recrystallization (19). While Schreiber Beach microfossils are well preserved, Mink Mountain assemblages (which have undergone higher diagenetic conditions) are more deteriorated (19). The Gunflint formation has been a target in the search for the oldest robust evidence of magnetofossils. Because of the particular magnetic properties of magnetofossils, experimental techniques in bulk macroscopic samples, such as first-order reversal curves (FORC) (8, 20, 21), have been frequently used as a strong indication of MT. Besides previous findings in magnetic extracts, studies have reported positive bulk magnetic signatures for magnetofossils in the Gunflint formation (22), but further microscopic analysis has suggested them to be false positives related to nanoscopic (tabular) iron-oxides within the interfaces of Fe–Mg carbonates (23).

The micrometric filamentous microorganisms reported in the Gunflint formation range within the same dimensions but bear distinct chemical/mineralogical compositions. Even so, these heterogeneities have been interpreted as the result of distinct alteration (24). In most altered cases, secondary hematite (*α*-${\text{Fe}}_{2}{\text{O}}_{3}$) is reported as coating these microfossil filaments (25). However, Ptychographic X-ray nanotomography, which is a synchrotron-based technique with unparalleled resolution at the nanoscale (26, 27), of samples from the Gunflint formation has revealed distinctive data (28). Quantitative three-dimensional electron density distribution of Mink Mountain fossils has shown filaments of 1 μm across, with electron densities matched as major structures of mature kerogen involving cuboid-euhedral crystals of maghemite [*γ*-Fe_2_O_3_, the low-temperature alteration product of magnetite (29, 30)]. More interestingly, bulk magnetic analysis of these same samples supports the interpretation of these cubic minerals as maghemite instead of hematite (28). As the oxidation of magnetite in Mink Mountain fossils might indeed have a diagenetic origin, it does not necessarily reflect the parental phase origin from a nonbiological process. The cuboid iron oxide particles found in Mink Mountain samples are intricately embedded within *Gunflintia* kerogen filaments. A majority of these particles appear misaligned within the linear organic filament structures, showcasing overgrowth due to diagenetic alteration. However, it is worth highlighting that the original morphology of the grains remains discernible, often exhibiting a consistent volume (approximately 1 μm long). This similarity in grain sizes is consistent with observations documented for Paleocene–Eocene giant magnetofossils (11).

To date, there have been no reports of giant magnetofossils exhibiting cubic particle morphology. Nevertheless, there is ample evidence demonstrating that modern MT can produce smaller cuboid grains. Notably, *Magnetospirillum* species, extensively studied among MT, biomineralize chains of cuboctahedral crystals of magnetite (31). Consequently, there is no compelling reason to dismiss the potential discovery of large magnetofossils with particles in cuboid-like morphologies. Despite the absence of conclusive evidence supporting the biogenic origin of these grains, the presence of kerogen in their surroundings might potentially indicate an origin from cell walls or magnetosome membranes. Together, the linear alignment of magnetic particles and the uniform morphology closely resemble the arrangement observed in contemporary magnetosomes.

The magnetic stability of modern magnetosomes is well known, but knowledge of giant magnetosomes and the biological function of synthesizing significantly large iron oxides (compared to the modern MT) remains poorly explored. Unlike the simple uniformly magnetized single-domain (SD) structures that characterize ordinary magnetosomes, larger biomineralized iron oxides will have more complex magnetic structures, from single (SV) to multivortex (MV) states. It is unclear whether magnetotaxis is possible for such organisms or if their biosynthesis serves a purely metabolic function. This paper reconstructs synthetic linear magnetosome chains using the morphology of grains in the collapsed chains seen in Mink Mountain fossils. Micromagnetic numerical models were then used to determine the likelihood that magnetotaxis is a possible navigational mechanism for giant magnetofossils.

## Results

### Micromagnetic Models.

The grains of maghemite, as imaged by Maldanis et al. (28), exhibit no rims or discernible variation patterns in electron density. The lack of these features and the elongated cubic morphology of the grains leads us to interpret the evidence as indicative of a homogeneous alteration process, specifically the transformation from maghemite to magnetite. Assuming that micrometric iron oxides represent remnants of giant magnetofossils, we expect that they initially formed a chain-like structure. The alignment of these grains within the linear filaments of organic matter stands as our primary supporting evidence for this assumption. Subsequently, we attribute the current arrangement to a combination of chain collapse (8) and additional diagenetic alteration processes. To conduct our modeling, we have used the most well-preserved grain morphologies within the filaments, specifically isolating a cuboid particle with an axial ratio of 1.26. Two chain alignment scenarios were tested: one along the axis of maximum elongation (designated as the X-axis) and another along the minimum elongation (designated as the Z-axis).

Given the assumption that these entities were originally organisms capable of biosynthesis, it remains uncertain whether they were multicellular or if membranes once separated the grains, similar to a magnetotactic bacterium. Despite this, we contend that such considerations are not pivotal in our speculative models assessing the potential for magnetotaxis in micrometric magnetite grains. In our analysis, the important factor is the separation of the magnetic particles. We assume a small distance between the grains (*≈*40 nm), sufficient to accommodate either a cell wall or intracell membrane. This is an approximation to the membrane thickness of ordinary magnetosomes (32, 33), up-scaled to micrometric grains. While multicellular organisms might be argued to have imposed larger boundaries between the magnetic particles, distances much larger than assumed in our models would have resulted in a much more significant *postmortem* particle clumping upon membranes’ degradation, and the observed linear chain of particles would not have been preserved.

Micromagnetic models were performed using MERRILL (34), an open-source code package that requires as input the 3D morphology of the particles as a discrete finite-element mesh and material properties of the magnetic mineral. Macroscopic magnetic and Ptychographic nanotomography data collected by Maldanis et al. (28) indicates maghemite as the composition. However, maghemitization is interpreted as a *postmortem* process due to diagenetic processes in which the rocks were exposed (9). Hence, we considered scenarios using properties of both maghemite and magnetite (with a 10% volume reduction to account for the mineral transformation).

We investigated the magnetic structure and stability of giant magnetosomes by finding the local energy minimum (LEM) within each particle. Two different classes of near-zero remanence domain structures were considered for the three-particle chain: I) states derived from random initial magnetizations in each particle and II) states derived from a uniform (saturated) initial magnetization parallel to the chain axis, implying different biomineralization processes. The random initial states relate to particles growing in distinctive regions of the organism that were subsequently brought into closer proximity. The saturated uniform guess assumes that particle growth has followed the organisms’ development, as the particles crystallize in a nanometric size already arranged in a chain structure and increase in volume up to our models’ dimensions.

**Fig. 1.**
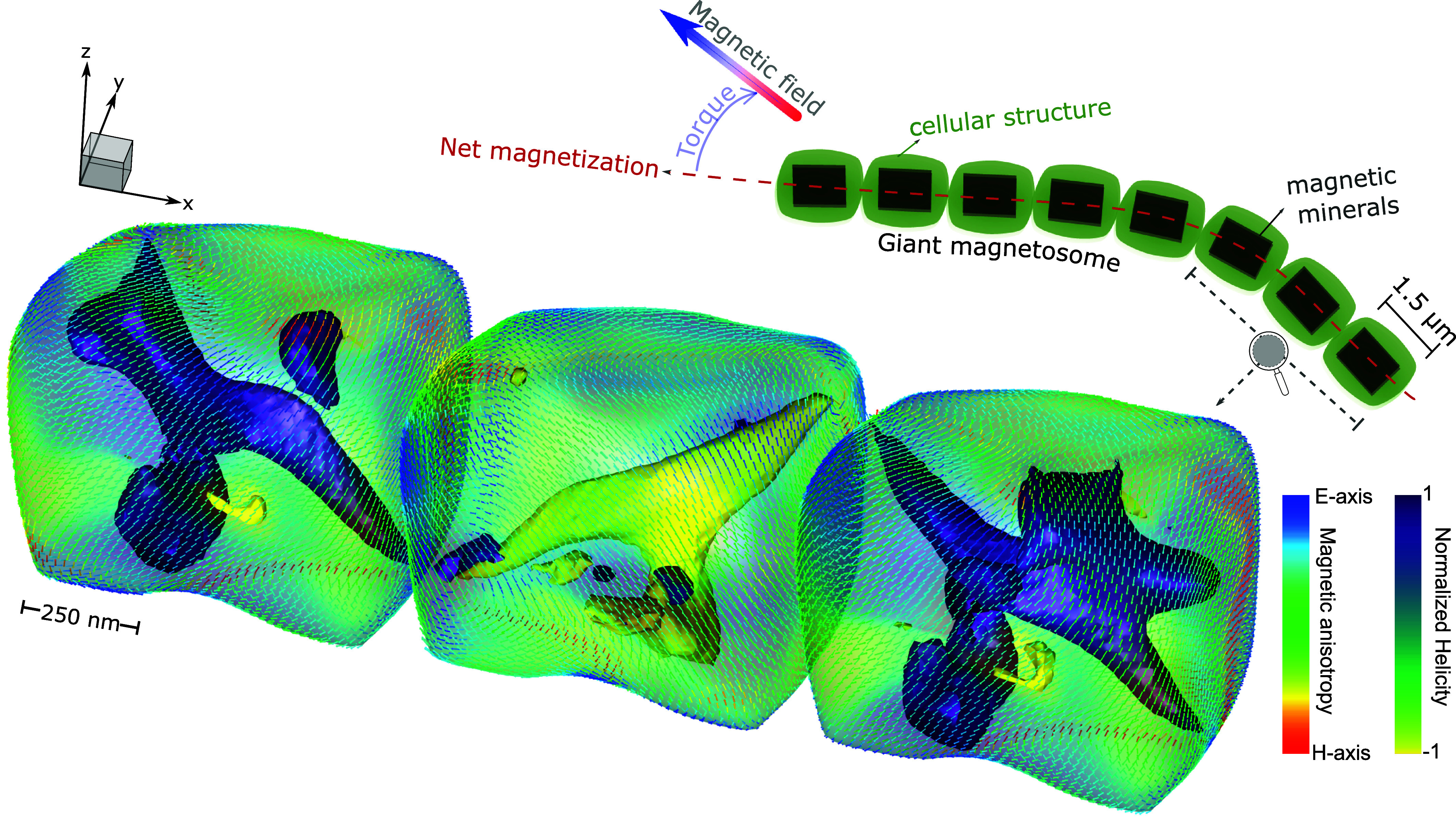
Minimization of the magnetic energy of a synthetic chain with three micrometric grains of magnetite aligned with maximum elongation axis. The arrows are hued by the magnetic anisotropy (blue tones for the easy axis and red for the hard axis). The vortices within each particle are the isosurfaces of the local helicities (which is the projection of the vorticity onto the magnetization). In the *Upper Right* corner, there is a schematic (speculative) representation of a giant magnetosome. Distinguishing from SD structures of modern nanosized chains of MT, micromagnetic models of giant magnetofossils converge to a multivortex structure.

The normalized helicity for all models (X and Z-aligned chains) showed nucleation of multiple vortices but with an average helicity alignment toward the chain axes in each grain (Fig. 1). The magnetization of grains in the vortex state, whether SV or MV, often referred to as the pseudo-single-domain (PSD), is typically dominated by the vortex core. In this state, the magnetic moment curls around the core, and this outer “shell” region is generally perpendicular to the core itself (35). Magnetite and maghemite exhibited similar MV morphology in both scenarios. Notably, magnetite chains aligned along the X-axis produced large vortex structures oriented with the [111] crystallographic axis.

**Fig. 2.**
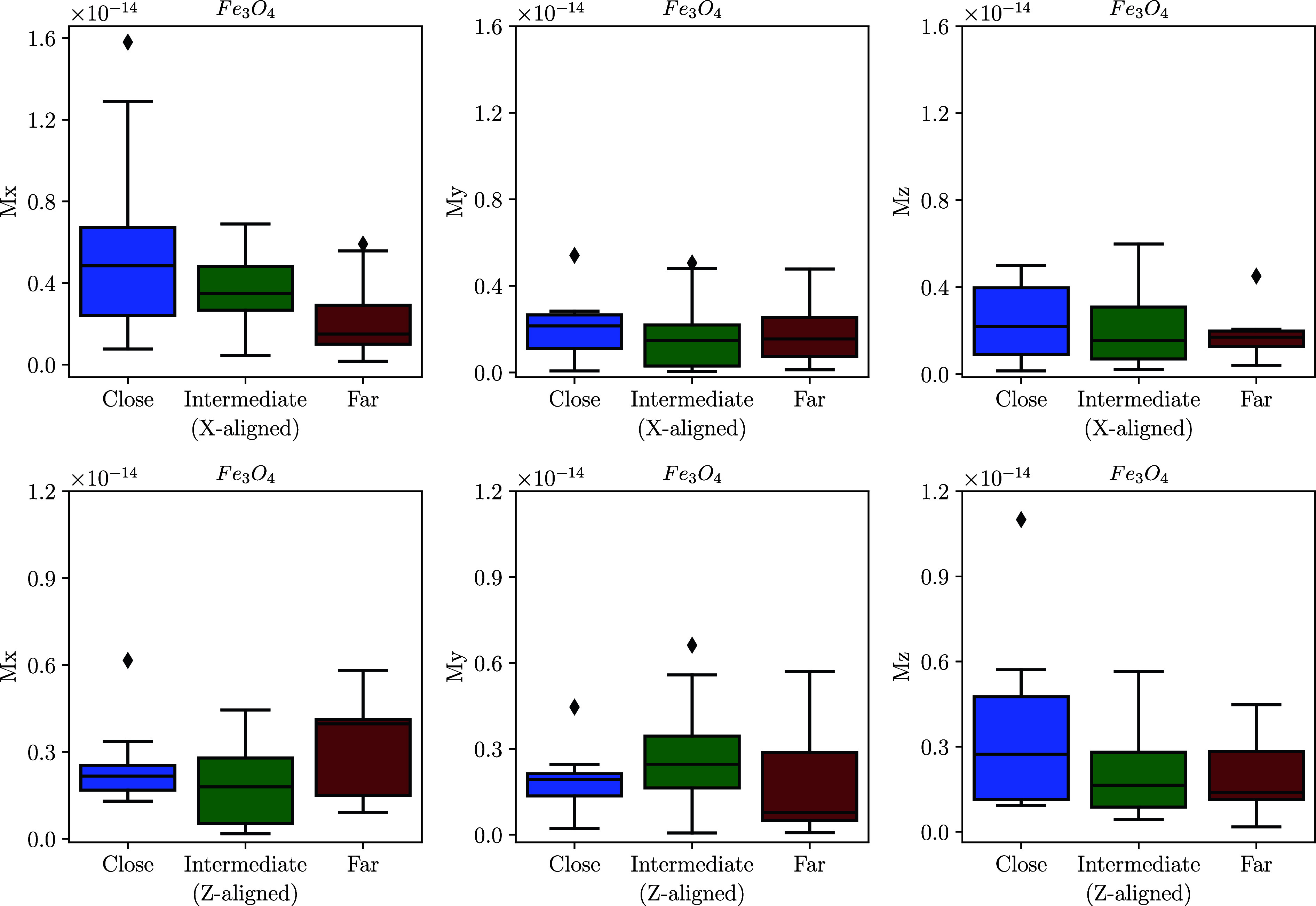
Magnetization of the three axes of the magnetite magnetosomes chains $\left(M_{x},\;M_{y},\;\text{and}\;M_{z}\right)$ changes as the interparticle distance is increased in random initial state scenarios. The first and second rows present the box plots for X/Z-aligned chains. These box plots demonstrate a consistently larger magnetization in the direction of the chain axis, which decreases with the strength of the magnetostatic interactions, as the magnetosomes are moved further apart. The diamonds in the box plots represent statistical outliers.

The arrangement of grains in a chain plays a crucial role in facilitating magnetotaxis, due to the effect of intergrain magnetostatic interactions. The effects are demonstrated in Fig. 2. When the spacing between particles in synthetic chains is varied from a few nanometers to a few microns (check *Materials and Methods* for clarity), the magnetization intensity decreases rapidly with increasing spacing, as expected. Considering the smallest interparticle distances in our simulations, for scenario (I) the net-magnetization of the three grains of magnetite resulted in total intensities of 7.33 *±* 4.22 f Am^2^ (or Mr/Ms=0.018±0.01); and $5.40\pm 2.98\;\text{f}{\text{Am}}^{2}$ (Mr/Ms=0.013±0.01), for X and Z alignments (respectively). For maghemite, it resulted in $5.70\pm 3.79\;\text{f}\;{\text{Am}}^{2}$ (Mr/Ms=0.013±0.01); and $4.70\pm 1.78\;\text{f}\;{\text{Am}}^{2}$ (Mr/Ms=0.011±0.004). The net-magnetizations calculated for the uniform initial guesses (scenario II) are not so different from the random ones, being $6.91/7.18\;\text{f}\;{\text{Am}}^{2}$(Mr/Ms=0.017/0.018) for magnetite, and $7.04/7.65\;\text{f}\;{\text{Am}}^{2}$(Mr/Ms=0.016/0.017) for maghemite. Thus even in these giant magnetosomes, magnetostatic interactions create a preferential alignment of the magnetization along the chain axis, which if sufficiently strong could enable magnetotaxis. In our numerical models, this is optimized for magnetite when magnetosomes are long-axis aligned (X-aligned) and for maghemite when it is short-axis (Z) aligned.

**Fig. 3.**
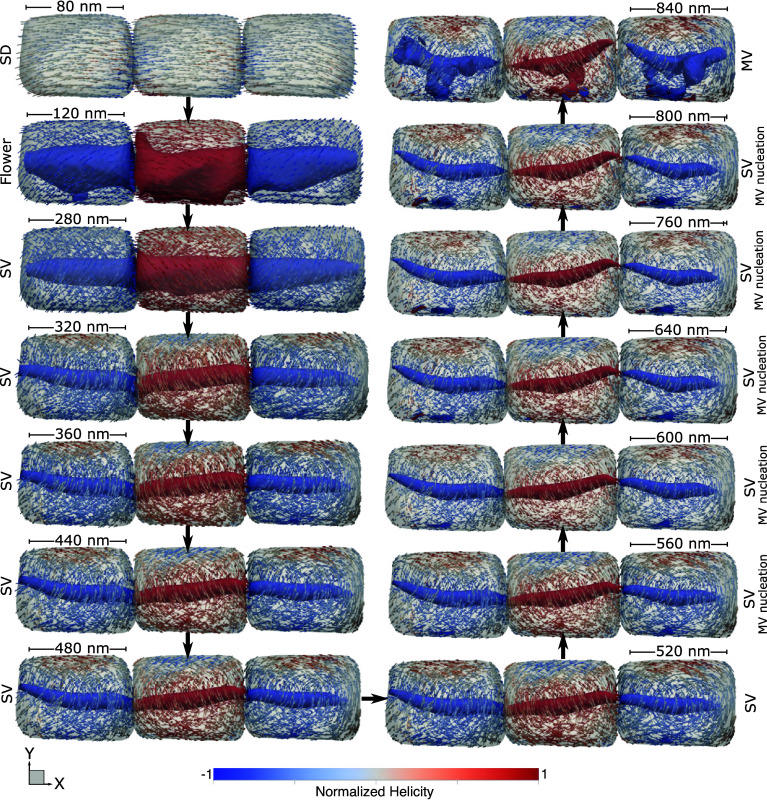
Evolution of the magnetic structure of a magnetosome of magnetite as a function of crystal growth, simulating the growth of an organism and a gradual increase in the particle size. Blue and red arrows represent the normalized helicity, and the general structure is more easily observed through their respective vortices (isosurfaces). The black arrows indicate the growing pathway. LEM calculations show that with the increase of particle size, the magnetic structure evolves from a uniform SD configuration to a nonuniform SV state. Multivortices start to nucleate for crystals with major axis *>*520 nm, but the configuration is majorly SV. The final MV state (which is our current size for the giant magnetosomes) only appears after 800 nm.

When we assume that particles slowly increase in size as they follow the organism’s growth, the evolution of one magnetic state to another is very consistent (Fig. 3). Initially, it passes from an SD state to more complex magnetic structures, first as SV structures and then to a MV structure. Yet, the magnetization is still consistently greater along the maximum elongation axis and produces a final magnetization even more substantial than a simple uniformed initial guess, also producing major helicity structures parallel to the [111] axis. A consistent MV state only appears for the largest grains in our simulations, which indicates that increasing the number of particles in the chain will also increase magnetic interaction. With an increase in magnetic interaction, the SV structure can maintain itself to even larger grain sizes and consequently increase remanence.

According to our simulations, the more consistent scenario is considering that these grains were originally magnetite that nucleated as expected in model II and oriented themselves along the X-axis (as in modern magnetotactic bacteria). Nevertheless, numerical models for chains with different major-axis orientations indicate that the resulting net-magnetizations are within the same magnitude.

**Fig. 4.**
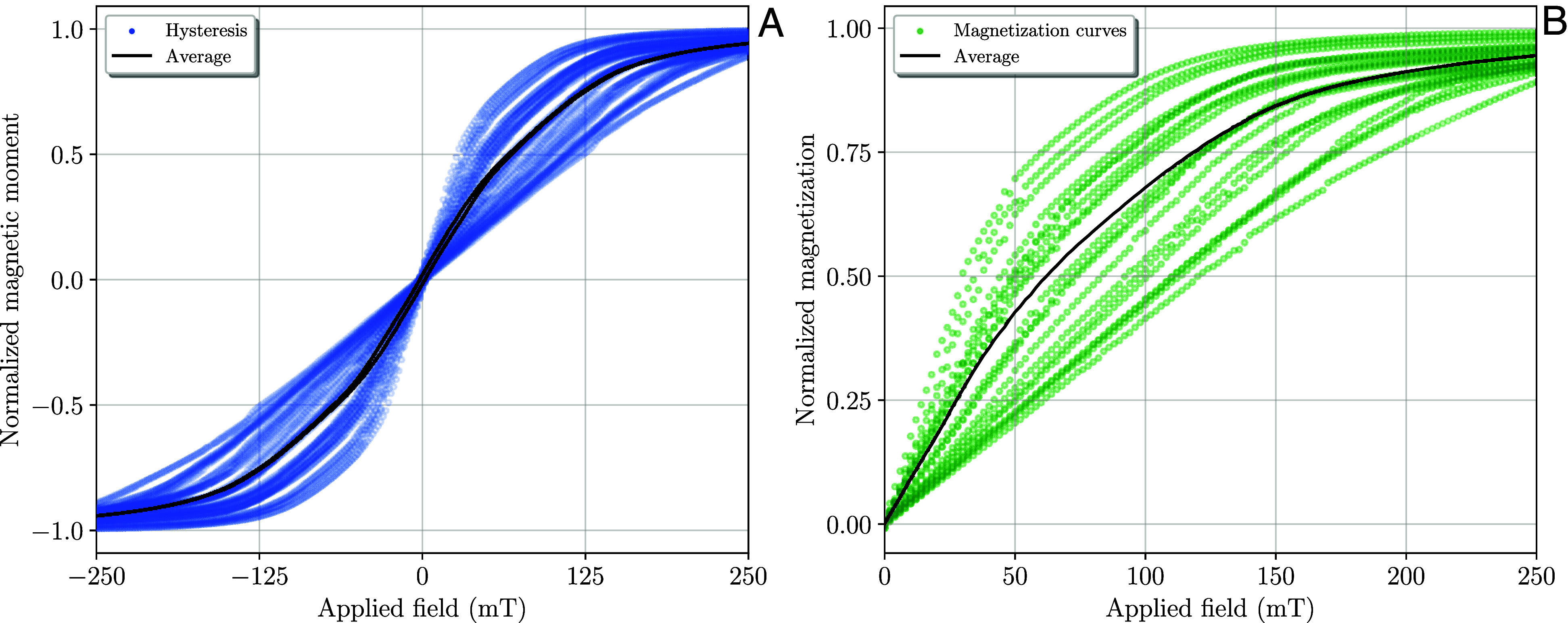
Magnetic hysteresis curves (*A*) and magnetization curves starting from random initial guesses (*B*) are shown for three-chained particles of magnetite aligned along the X-axis. The models were run over 20 different random field directions. Although the magnetic hysteresis curve shows a small coercivity, the magnetization curves starting from random initial guesses demonstrate considerably high destructive in-fields, resulting in a bimodal population with two distinct magnetic distributions: one soft and the other hard. This bimodal distribution arises as an effect of the anisotropy of the chain.

Simulated magnetic hysteresis determined from averages of 20 different field directions resulted in thin-waisted loops with coercivities of 2 mT in all scenarios for the X-aligned particles (Fig. 4*A*). This low coercivity is notably different from that observed in SD-like chains of MT, where the coercivity populations (in FORC diagrams) are centered around values of at least 40mT (8, 20, 21). However, the coercivity value of 2mT would marginally increase with the number of magnetosomes in the chains, where this lower coercivity limit is still at least an order of magnitude much larger than the Earth’s magnetic field strength. Distinguishing from the hysteresis that starts from a uniform saturated state, when we calculate in-field magnetization curves starting from random initial guesses, the curves exhibit a bimodal distribution of increments (Fig. 4*B*). The first mode shows a lower coercivity, acquiring most of the magnetization before reaching 50 mT, which is a common behavior for magnetite. The second mode displays higher coercivity, peaking near 80 mT. This dual behavior is a response to the uniaxial anisotropy of the chain.

**Fig. 5.**
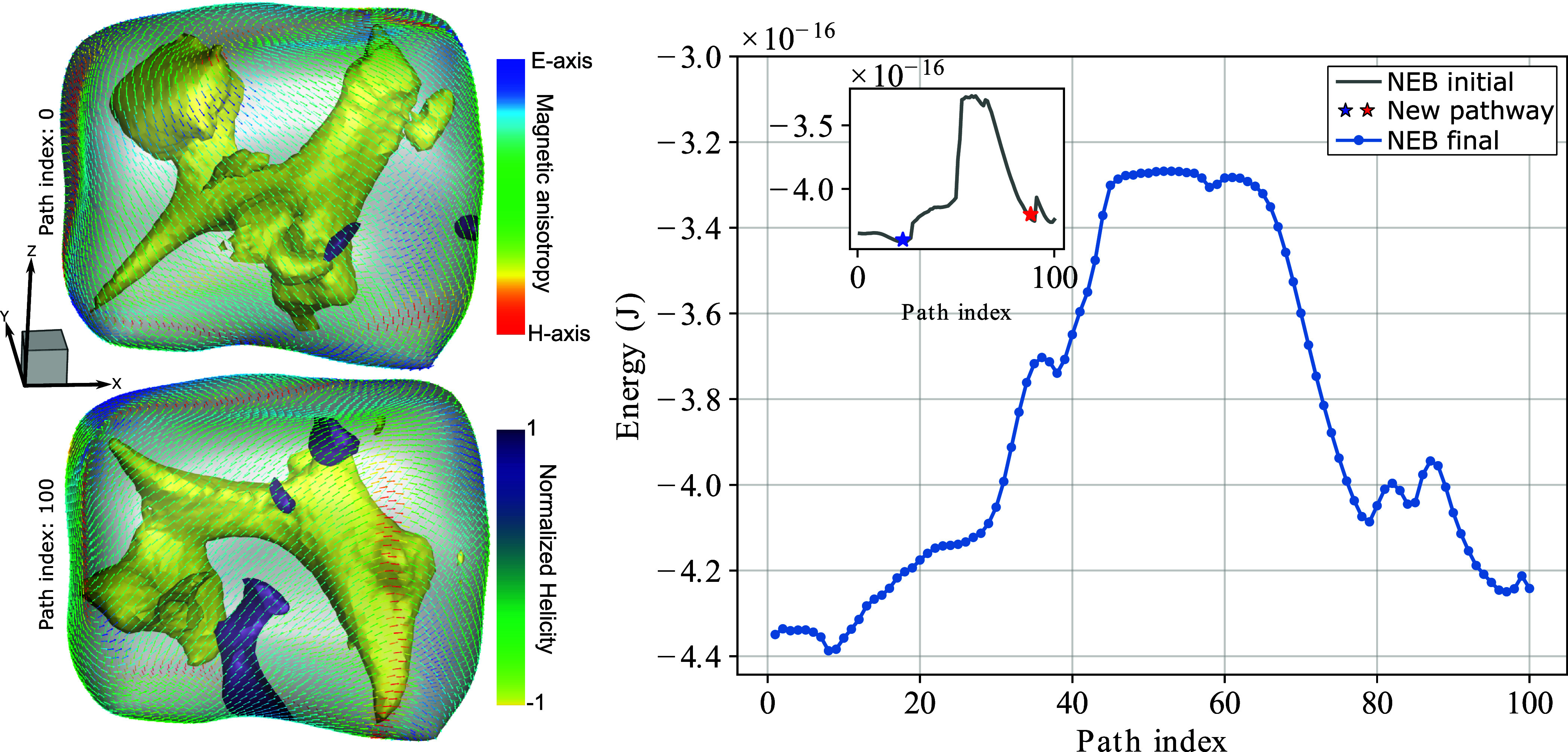
Calculation of the magnetic energy barrier between two opposite magnetic states of a micrometric grain of magnetite (refer to the structures on the left side of the figure, Path index 0 and 100, respectively). Initially, a calculation is performed using the nudged elastic band (NEB) method (35) to identify local minima (represented by blue and red stars). Subsequently, a final minimization is conducted between these two states (NEB final). The presence of an enormous energy barrier indicates that such a multivortex state characterizes highly stable magnetic particles. The grains’ scale is the same as in Fig. 1.

To ensure the stability of a large magnetosome structure, we performed calculations to determine the required thermal energy for a magnetic state domain (in a single particle) to undergo a 180^°^ rotation (a mirrored state). Using this information, we accessed the relaxation time (τ) of one of these particles, which represents the time required for thermal energy to randomize the magnetic moments within the particle (Fig. 5). The calculation revealed that such individual grains are stable over geological times, with domain state relaxation times greater than the age of the Solar System. The magnetostatic interactions present when such grains are arranged in a chain would only further increase the thermal and temporal stability.

### Magnetotaxis Efficiency.

Besides showing that such giant magnetosomes of magnetite can interact to produce a larger magnetization within their alignment axis, the net-magnetization produced by the magnetosome chain must be sufficiently large for its torque toward the environmental field to enable efficient magnetotaxis. For a given population of cells forming an organism, the average alignment (⟨cos(θ)⟩, where *θ* is the angle between the field direction and the magnetization of the organism) is a function dependent on the strength of the magnetic torque produced by the chain and the thermal energy of the fluid’s Brownian motion. Some authors (36) consider that to offer any biological advantage to a magnetotactic organism, ⟨cos(θ)⟩≥0.5. Even in a basic model containing only three particles under a weak magnetic field of 6 μT, the alignment condition is fulfilled in all of our simulations. The substantial volume of iron oxides in giant magnetofossils results in a higher magnetization compared to modern MT, which necessitates longer chains to meet the alignment requirement. A better way to evaluate the efficiency of magnetotaxis is analyzing whether it allows faster displacement over a given distance compared to relying solely on chemotaxis for the same displacement. Mao et al. (6) have demonstrated that even small alignments are advantageous when the traveled distance is sufficiently large. However, they also showed that alignment in sediment pores is much less efficient compared to MT performing magnetotaxis in the water column. As we cannot determine the living environment, we can simply compare the Brownian alignment in a low magnetic field (as stated above) to be as efficient as that of modern MT (e.g., *Magnetobacterium bavaricum*) (6) and magnetotactic holobionts (36).

Nevertheless, possessing the necessary energy to produce an alignment is not enough to guarantee efficient magnetotaxis. The time that the organism takes to turn toward the field can be an important constraint. For instance, we can calculate the necessary time for an organism to perform a full $180^{{}^{\circ}}$ turn upon the polarity switching of a magnetic field (U-turn) (37). An actual geomagnetic field reversal occurs on a much larger time scale compared to the lifespan of a magnetotactic organism. Instead, the U-turn here represents episodic disturbances in the living environment or the mechanical movement of an organism (such as swimming) that mismatches the direction of the geomagnetic field. The U-turn motion depends on the: i) magnetization produced by a giant magnetosome; ii) its morphology/dimensions; iii) the physical properties of the medium and; iv) the intensity of the environmental magnetic field. Often models related to the torque of a magnetic particle in a fluid assume a spherical morphology for the particles (38–41). However, a more realistic model should account for the ellipsoidal shape of a magnetotactic organism. The viscous torque of a prolate ellipsoid (whose main axes are a, b, and c) rotating in a uniformly magnetized fluid (42) can be assimilated to the U-turn motion approach (36, 37) to produce a modified model that allowed us to calculate the hypothetical times for giant magnetofossils.

**Fig. 6.**
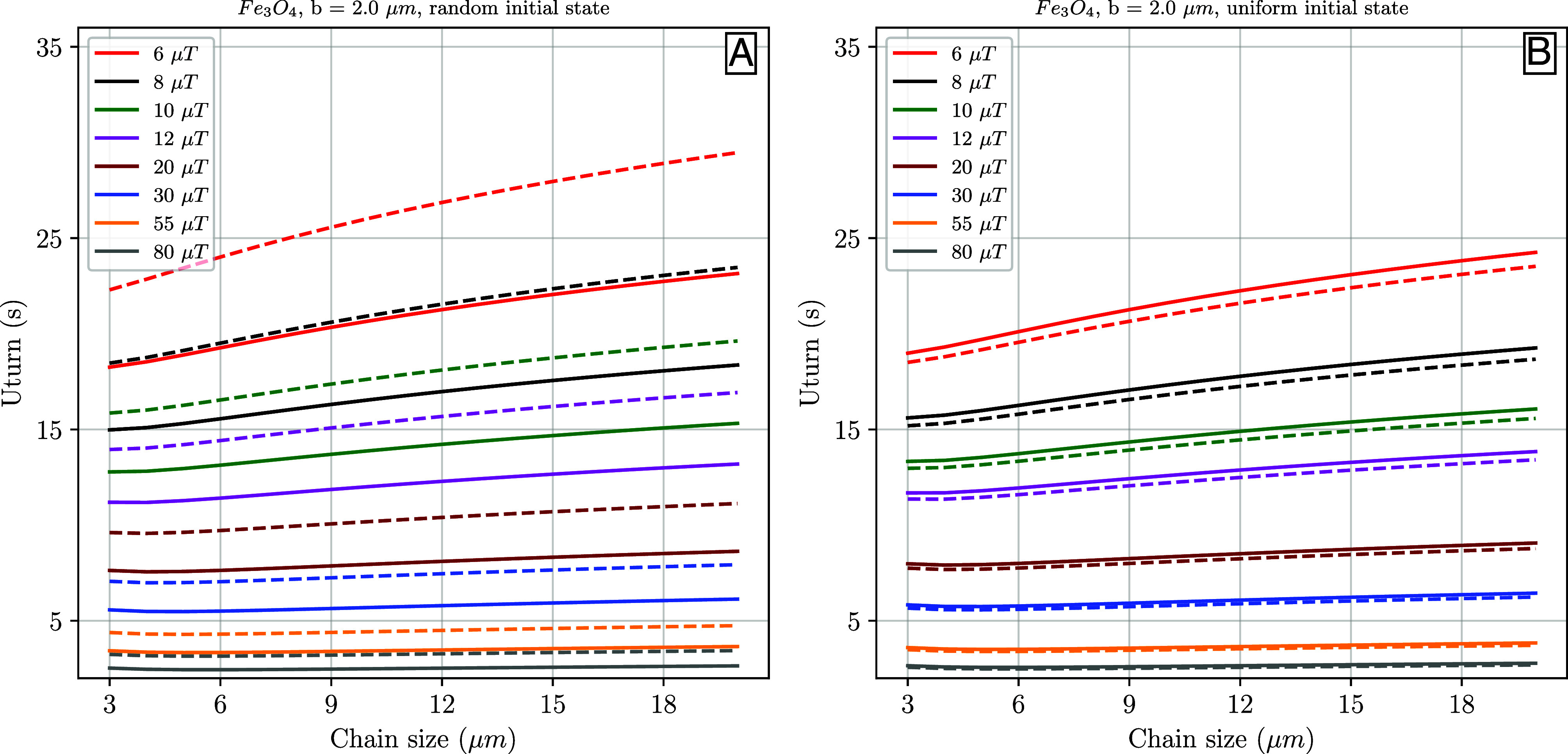
The required time for the giant synthetic magnetite chains to rotate due to a polarity switching field (U-turn motion) is determined. The calculations utilize the net-magnetization obtained from numerical models, considering both (*A*) a random initial state and (*B*) an uniform initial state, for different field intensities and chain sizes. Solid lines represent X-aligned chains of magnetite, while dashed lines represent Z-aligned chains. *b* = 2 μm refers to the minimum elongation axis of the giant magnetosome, while the chain size (X-axis) represents the maximum elongation (called *a*, visit U-turn motion within the *Materials and Methods* section).

We use the net-magnetization intensities acquired from the micromagnetic models to calculate the U-turn motion for our synthetic chains of giant magnetofossils (Fig. 6). As we cannot determine the number of particles originally composing these chains, we perform simulations to calculate the torque of a bacterial body in water, of length 3 ≤ *a* ≤ 20 μm (containing a single magnetic chain of 3 to 20 particles) under ambient field intensities varying from 6 to 80 μT. The same uncertainty remains on the size of the b-axis of the bacterial body, but we assume these to be at least twice the diameter reported by Maldanis et al. (28) (*b* = 2 μm), to account for cellular structure enveloping the particles. Using either the average magnetization acquired from scenario I or the uniform state magnetization from II, the general behavior of the U-turn curves are comparable. Although the net-magnetization of the bacteria body increases linearly with magnetosome chain length, this also means that magnetic energy must overcome an increasing influence of the Brownian motion as the chains become longer. This effect is especially important at low magnetic fields, as the U-turn times are more sensitive to chain length (Fig. 6). For fields higher than 15 μT, magnetic energy is sufficiently large to overcome the thermal energy and efficiently rotate the particle toward the field in practically the same amount of time, independent of chain length.

U-turn motion times for magnetite (aligned along X or Z) in the presence of weak and strong environmental magnetic fields exhibit comparable durations for both scenarios I and II. In the case of an intermediate-size chain (*a* = 12 μm) of a giant magnetofossil in a weak magnetic field (6 μT), the U-turn takes approximately 21/22 s (in scenarios I/II for X-aligned chains). However, when the magnetic field intensity approaches the averaged (present) geomagnetic field strength (55 μT), the turn times decrease to around 3/4 s. Conversely, for maghemite, average magnetizations (in scenarios I/II for X-aligned chains) in worst-case/best-case scenarios resulted in durations of approximately 25/23 s and 3 s, respectively.

U-turn motion experiments for several different kinds of modern magnetotactic organisms (39), such as magnetotactic cocci (43, 44), have shown similar magnetic moments to those we have modeled, even when we consider only three grains. For modern large flagellated protists (*≈*23 μm) (37), the magnetic moment estimated from U-turn times, with a much higher magnetic field intensity, can be one order of magnitude larger than that of ordinary MT. However, these protists contain multiple chains of bullet-shaped magnetosomes in the cell. In summary, our numerical magnetic models imply that giant chains formed by micrometric iron oxides yield the properties of stable magnetic structures and can produce magnetotaxis even at low magnetic field intensities, regardless of their original orientation or mechanism of crystal growth.

## Discussion

The existence of giant magnetofossils raises questions regarding the biological role in synthesizing such large grains. As of now, particles of giant magnetofossils have been found dispersed throughout the sediments, rather than forming chains (7, 11). This distribution potentially suggests a protective function rather than being associated with magnetotaxis (11). Chang et al. (7) explored different morphologies of giant magnetofossils (spearhead and needle-like) using micromagnetic models and proposed that magnetotaxis might be dependent on particle size and shape. Our study focuses on cuboid-like particles in a micrometric chain structure. Contrary to conventional assumptions, we quantitatively demonstrate that magnetotaxis is possible even with more complex magnetic states like multivortex configurations, providing important insights into this phenomenon.

The signatures of these particles, however, could be hard to spot in bulk magnetic analysis. Wagner et al. (45) argue that it is possible to distinguish conventional magnetofossil chains from giant needle-shaped particles when applying principle component analysis in FORC diagrams. That seems adequate for such a specific morphology of giant grains spread in a matrix, but probably not entirely adequate for other morphologies such as cubic-like habits (as explored in our work). Contrasting the known magnetic signatures of ordinary MT [e.g., the central ridge in FORC diagrams (21)], giant magnetofossils (in morphologies of spearhead, needle, or cubic-like particles) have not been yet reported forming chain structures. Even if chain structures of giant magnetofossils are present in sedimentary rocks, their collapse might still result in small net-magnetization and minimize the along-axis magnetic interaction of the particles (8). This makes it challenging to associate bulk magnetic measurements with the presence of these giant magnetofossils, given the distinct magnetic properties arising from their larger dimensions. We, therefore, suggest that presently the most reliable method to find remnants of giant chains is to employ 3D imaging techniques that do not require magnetic extraction and are sensitive to chemical/structural composition at the micro- and nanoscales.

The geological record of giant magnetofossils is scarce. Some known examples are the *≈*56 million years old sediments from the New Jersey coastal plain (46) and from the Southern Ocean near Antarctica in a similar time interval (7). Initially, their limited occurrence during the Paleocene–Eocene Thermal Maximum led to the hypothesis that changes in the biogenic cycle of iron caused the enlargement of suboxic/anoxic zones, promoting the biosynthesis of these magnetofossils (11, 45, 46). However, discoveries of such organisms earlier (*≈*700 thousand years) and later (*≈*300 thousand years) than the Paleocene–Eocene boundary expand their geological timescale and thus might decouple the paleoclimate implications (47). Rather, the occurrence of giant magnetosomes seems to be limited (majorly) by specific redox conditions and an excessive amount of dissolved iron.

Because both ordinary magnetosomes and giant spearhead/needle-like magnetofossils have been found in the same Paleocene/Eocene sediments, it is unlikely to single out the gigantism of these specific magnetofossils as a primordial evolutionary feature, especially because there is evidence of ordinary magnetofossils spanning up to 1.9 Ga (5). However, in the context of the organic filaments found in Gunflint fossils, the presence of micrometric cubic grains could suggest the potential existence of a primordial organism capable of producing magnetosomes with distant evolutionary ties to modern MT. Distinguishing from the well-preserved Schreiber Beach filaments, Mink Mountain filaments are very often described as poorly preserved due to the rocks’ diagenetic conditions (19). However, the use of an advanced in situ tomographic (28) approach allowed the verification of both organic matter and the occurrences of maghemite iron oxides systematically associated with them in the samples of Mink Mountain. The iron existing in the banded iron of the Gunflint formation is predominantly derived from hydrothermal vents, where the up-welling of Fe-rich deep water brings nutrients to more superficial waters (48). Fe-isotope studies in stromatolites from the Gunflint formation suggest formation in low oxygen conditions. This implies the presence of a redoxcline in shallow water depths, providing evidence for the existence of Fe-oxidizing bacteria (17, 48). Therefore, it is reasonable to consider that the environmental conditions present during the Gunflint formation were conducive to the emergence of giant magnetofossils observed thus far.

Searching for primordial life forms in ancient geological formations is a vital task in building evolution trends of life on Earth. However, it is also essential to understand the environmental conditions where life developed and further evolved. Despite the combination of phylogenetic and molecular biology techniques used to “resurrect” ancient proteins to gather paleoenvironmental insights (49, 50), most of our understanding of Precambrian environments is achieved from geological/geochemical proxies. Distinguishing from “conventional fossils,” the presence of magnetofossils reflects important redox conditions that allow magnetotactic bacteria to grow and be preserved, which makes them a very frequently unexplored indicator for ancient biochemical processes (9). Since molecular clock dating methods suggest that the emergence of MT occurred during the Archean (12), it is crucial to identify these organisms in the fossil record. This is especially important because their presence is relevant to Earth’s biogeochemical iron recycling cycle. Furthermore, the period when MT are believed to have appeared in the Precambrian aligns with significant global changes in oceanic redox conditions and the formation (51) of banded iron formations. From this perspective, the magnetic properties of rocks, which are fast and cost-effective techniques sensitive to even traces of magnetic particles, can be of essential aid, especially when combined with microscopy. In particular, the use of nondestructive high-resolution imaging methods [such as Ptychographic nanotomography, (26)] to identify morphological biosignatures in rocks might be an important tool in the search for microbial life, not only on Earth but also in extraterrestrial samples. Furthermore, by pairing these imaging methods with micromagnetic modeling of three-dimensional morphological data of magnetofossils, we can gain insights into the biological function of Fe-biosynthesis in early forms of life.

## Materials and Methods

### Data Preparation.

To prepare the synthetic chains investigated in our numerical simulations we use the Ptychographic X-ray nanotomography reconstructions of the *δ*-phase data (Mink Mountain sample) acquired by Maldanis et al. (28) in the cSAXS beamline of the Swiss Light Source. Specifically, we work with segmented data that corresponds to the maghemite spectrum in the electron density distribution $\left(1.40\;e^{-}\cdot {Å}^{-3},\sigma_{1}=0.03\right)$. These data were loaded on the software Autodesk Fusion 360, where the morphology of the cubic-like grain was selected and manually segmented from the magnetofossils, resulting in a particle with a maximum/minimum elongation axis of 0.91/0.72 μm. We have replicated this grain morphology twice to form the synthetic chains, aligning them in a chain-like structure along their maximum/minimum elongation axis (X/Z-axis) keeping a separation of *≈*40 nm (since the morphology of the grains is not entirely regular). These data were then exported as stereolithographic data and read into the software Coreform Cubit to generate the 3D-finite element meshes necessary to perform the micromagnetic calculations. Because we expect maghemite to expand in 10% in volume, we have reduced the original volume for the same amount when producing meshes for magnetite.

Micromagnetic models usually assume that the mesh is fine enough to resolve the spatial variation magnetization within the morphology, which usually requires maximum element sizes that respect the “exchange length, $I_{\mathrm{exch}}$” (52). In environmental temperature, $I_{\mathrm{exch}}$ is 9 nm for magnetite (34). However, our simulations of these giant magnetosomes include three micrometric particles, which would require an unreal processing time. Instead, we have produced linear tetrahedral meshes using an interval sizing slightest larger (20 nm), in a way to maintain a still reliable solution. This is quite a standard compromise (53–55) to achieve a model feasible for the micromagnetic algorithm and available computational resources. The change from a 9 nm to a 20 nm mesh will not affect the predicted magnetic domain structure, but there might be a slight underestimation of magnetization in highly in-homogeneous magnetic structures. Consequently, our models establish a lower limit for magnetotaxis efficiency, without compromising the robustness of our conclusions.

These meshes were used in the calculation of local energy minima (LEM) for scenarios (I, random initial guesses) and (II, uniform initial guesses), magnetic hysteresis, and magnetization curves starting from random initial guesses. In the case of energy barrier calculations performed in a single grain, a more refined mesh could be applied (16 nm). Finally, we employed a distinct configuration to calculate models for growing chains of magnetite. This approach involved generating 20 diverse meshes, where each mesh contained individual particles ranging from 0.086 μm (with a 9 nm interval sizing) to 0.823 μm (with a 20nm interval sizing). Increasing the particle sizes within the chain and observing their progression serves as a representation of the development and growth of an organism. During the early-life stages, we assume that not only are magnetic grains smaller, but the interparticle separation is also proportionally reduced. The interval sizing was adapted based on the size of the chain (check *Data, Materials, and Software Availability* for the link where you can find more information about this procedure), by performing a scale adjustment simultaneously to the entire system (the three grains), with alterations to their separation also adhering to the same scale.

### Numerical Models.

For the calculation of three-dimensional micromagnetics, we have used the open-source package MERRILL (34). The properties of the magnetic minerals in our simulations assume standard conditions of temperature and pressure. For magnetite $\left({\text{Fe}}_{3}{\text{O}}_{4}\right)$, they are the I) exchange constant $\left(A_{\text{ex}}=1.33\cdot 10^{-11}\;{\text{Jm}}^{-1}\right)$; the magnetocrystalline anisotropy constant $\left(K_{1}=-1.24\cdot 10^{4}\;{\text{Jm}}^{-1}\right)$; and the saturation magnetization $\left(M_{s}=4.8\cdot 10^{5}\;{\text{Am}}^{-1}\right)$ (56). For maghemite (*γ*-${\text{Fe}}_{2}{\text{O}}_{3})$, the same properties are $A_{\text{ex}}=1.00\cdot 10^{-11}\;{\text{Jm}}^{-1}$, $K_{1}=-4.60\cdot 10^{3}\;{\text{Jm}}^{-1}$ and $M_{s}=3.80\cdot 10^{5}\;{\text{Am}}^{-1}$ (57). Visualization of the minimizations was performed through Tecplot, by first computing the vorticity $\left(\vec{\omega }\right)$ as (Eq. **1**):

[1] $$\begin{array}{c} \vec{\omega }=\nabla \times \vec{M}, \end{array}$$

where $\vec{M}=M_{x}\hat{i}+M_{y}\hat{j}+M_{z}\hat{k}$. Finally, the normalized helicity $\vec{H}$ is (Eq. **2**):

[2] $$\begin{array}{c} \vec{H}=\frac{\vec{M}\cdot \vec{\omega }}{\left|\right|\vec{\omega }\left|\right|}. \end{array}$$

When dealing with LEM minimizations for random initial guesses (scenario I), 10 models were run for each of the configurations (maghemite/maghemite, X/Z aligned). That is necessary because, contrary to the uniform initial guesses, random moments might result in slightly different final solutions. The rescaled magnetization intensity of each of the scenarios $M_{\mathrm{sc}}$, which will be further used when calculating the U-turn motion times, is computed as (Eq. **3**):

[3] $$\begin{array}{c} M_{\mathrm{sc}}=M\cdot M_{s}\cdot V, \end{array}$$

where *V* is the volume of the particle and $M=\left|\right|\vec{M}\left|\right|$. The magnetization intensity verification along the main axes for synthetic particles was also performed by measuring the magnetization at different interparticle distances. The particles were separated to an intermediary distance (≈150 nm) and a long distance (*≈*2,700 nm), and then the magnetization intensity was computed as in Eq. **3**, changing the magnetization therm for $M_{x}$, $M_{y}$, and $M_{z}$ (respectively).

For both magnetic hysteresis and magnetization curves starting from random initial guesses, it is necessary to perform simulations in variable field directions to account for the magnetic anisotropy of the grains. We perform these analyses in 20 different directions drawn from a Fibonacci random sequence on the surface of a sphere, from *−*250 to 250 mT (in 2 mT steps), and average the final normalized magnetic moment (m) as (Eq. **4**):

[4] $$\begin{array}{c} m=\frac{\left(M_{x}\cdot B_{x}\right)+\left(M_{y}\cdot B_{y}\right)+\left(M_{z}\cdot B_{z}\right)}{M_{s}\cdot V}, \end{array}$$

where $B_{x}$, $B_{y}$, and $B_{z}$ are the applied field in those respective directions. To assess the energy barriers and confirm the stability of the grains forming the chain, we examined the magnetic structure of the growing chain models. Specifically, we focused on the state of the largest volume within the growing chain model. By rotating this state by $180^{{}^{\circ}}$, we obtained two opposite domain states, allowing us to analyze the magnetic energy $\left(E_{m}\right)$ required to transition from one state to the other using the NEB method (35) through MERRILL. Due to the irregular shape of the particle, the rotation results in eventual regions without an assigned magnetic moment, which was solved by filling these spots with random moments (to avoid disturbing the solutions). Models using initial guesses may lead to finding local minimum/maximum points in the energy profile. Then, we determine the lowest energy state among these initial guesses and proceed to recalculate the energy between the points of local minima. Finally, we can calculate the relaxation time (τ) of an individual grain as (Eq. **5**) (57):

[5] $$\begin{array}{c} \tau =C\cdot \exp\left(\frac{E_{m}}{E_{T}}\right), \end{array}$$

where $C=10^{-9}\text{s}$ is a frequency factor and $E_{T}$ is the thermal energy ($K_{B}\cdot T$, the Boltzmann constant $1.3806\;\cdot \;\;10^{-23}\;{\text{m}}^{2}\cdot \text{kg}\cdot {\text{s}}^{-2}\cdot {\text{K}}^{-1}$ and the temperature 293.15 K, respectively).

### U-turn motion.

In MT examples, we can better determine the number of magnetic particles forming a magnetosome and more easily perform a calculation of the total net-magnetization. For ancient organisms bearing magnetotaxis, the total extension of these organisms and the number of particles is speculative. Because our numerical models include only three particles in the simulation, we can assume that the net-magnetization $\left(M_{n}\right)$ produced by an organism of variable with variable chain size is (Eq. **6**):

[6] $$\begin{array}{c} M_{n}=\frac{M_{\mathrm{sc}}}{3}\cdot N, \end{array}$$

where *N* is the hypothetical number of particles in the chain and the divisor (3) arrives from the size of the synthetic chains used in the simulations. Sequentially, we can calculate the average alignment efficiency (⟨cos(θ)⟩) using $M_{n}$, for any number of *N* particles as (Eq. **7**) (39):

[7] $$\begin{array}{c} \left\langle \cos\left(\theta \right)\right\rangle =\;\coth\left(\frac{M_{n}\cdot B}{K_{B}\cdot T}\right)-{\left(\frac{M_{n}\cdot B}{K_{B}\cdot T}\right)}^{-1}, \end{array}$$

where *B* is the intensity of the magnetic field (T). Finally, the variable U-turn motion of a given prolate-shaped organism under the influence of a magnetic field can be calculated as (Eq. **8**) (37):

[8] $$\begin{array}{c} \tau_{\text{U-turn}}=\frac{A}{M_{n}\cdot B}\cdot \ln\left(\frac{2\cdot M_{n}\cdot B}{K_{B}\cdot T}\right), \end{array}$$

where $M_{n}$ is the net-magnetization $\left(A\cdot {\text{m}}^{2}\right)$, and A is a magnetoviscous coefficient adapted from Stiles and Kagan (42), written as:

[9] $$\begin{array}{c} A=\left|\left(\frac{16}{3}\right)\cdot \pi \cdot \eta \cdot c^{3}\cdot {\left(\frac{1}{2}\cdot \ln\left(\frac{a+c}{a-c}\right)-\frac{a\cdot c}{b^{2}}\right)}^{-1}\right|, \end{array}$$

where *η* is the dynamic viscosity of water $\left(10^{-3}\text{N}\cdot \text{s}\cdot {\text{m}}^{-2}\right)$; a/b are the maximum/minimum elongation axes and $c=\sqrt{a^{2}-b^{2}}$.

## Data Availability

All of the data related to this research are preserved at https://doi.org/10.5281/zenodo.8390679 and can be publicly accessed from the same repository. The data are subdivided into folders, including the meshes used in our simulations (.pat files), Python scripts, MERRILL scripts, and outputs achieved from the simulations (.out files). The results reported here were achieved through MERRILL (34), an open-sourced code for micromagnetic modeling of tetrahedral finite element meshes. Installation, guide tutorials, and courses are available at https://blogs.ed.ac.uk/rockmag/.
